# Home initiation of apomorphine infusion: lessons from the COVID-19 pandemic and implications for current clinical practice

**DOI:** 10.1007/s00702-023-02710-w

**Published:** 2023-10-23

**Authors:** Christopher Kobylecki, Lucy Partington-Smith

**Affiliations:** 1 Department of Neurology, Manchester Centre for Clinical Neurosciences, Northern Care Alliance NHS Foundation Trust, Stott Lane, Salford, M6 8HD UK; 2 Division of Neuroscience and Experimental Psychology, University of Manchester, Manchester, UK; 3 Department of Neurosurgery, Manchester Centre for Clinical Neurosciences, Northern Care Alliance NHS Foundation Trust, Salford, UK

**Keywords:** Parkinson’s disease, Apomorphine infusion, Clinical practice

## Abstract

Starting Parkinson’s disease (PD) patients on subcutaneous apomorphine (APO) infusion is generally undertaken on a hospital day-case basis. During the COVID-19 pandemic, day-case facilities were unavailable. To avoid delays in treatment, a new procedure was developed for initiation of APO therapy in the patient’s home. A home initiation protocol was developed and followed for each patient in this analysis. The hospital team worked in collaboration with APO nurses provided by the manufacturer of APO therapies to implement initiation and undertake follow-up. In this analysis, 27 PD patients were initiated onto APO infusion and 21 (77.8%) achieved a therapeutic response. Home initiation of APO infusion can be undertaken successfully and has benefits for both patients and healthcare teams. This protocol will now continue as a standard of care at our centre.

## Introduction

Apomorphine (APO) infusion is a potent dopamine receptor agonist with an established role for the treatment of motor symptoms of Parkinson’s disease (PD), including tremor, bradykinesia, and rigidity (Muller 2020). APO is administered subcutaneously as either a pen injection or a continuous infusion. The injection can be used as an ‘on demand’ therapy for the management of OFF episodes as an adjunct to oral medication, whereas APO infusion is indicated when PD has advanced to the stage when the person is experiencing persistent motor fluctuations that can no longer be effectively controlled with optimized oral medication (APO-go PFS EU Summary of Product Characteristics 2018). The infusion is administered using a small, wearable pump, and provides continuous dopaminergic stimulation which is known to be better at controlling motor fluctuations than intermittently administered oral/injectable medication (Olanow et al. 2020). In a recent randomized, double-blind placebo-controlled trial, APO infusion proved to be effective at reducing OFF time in patients with advanced PD and provided a corresponding increase in ‘good’ ON time without troublesome dyskinesia (Katzenschlager et al. 2018), and these beneficial effects persisted with long-term use (Katzenschlager et al. 2021).

APO infusion has been used successfully in clinical practice use for the management of PD for over 30 years and is generally well tolerated by patients (Jenner and Katzenschlager 2016). Of the available device-aided therapies for PD, APO infusion is generally considered as the least invasive as it does not require surgery. In the UK, APO is recommended by current National Institute for Health and Care Excellence (NICE) guidelines as part of the best medical therapy and before surgical options are considered, such as deep brain stimulation or the insertion of a percutaneous endoscopic gastrostomy (PEG) tube for intestinal levodopa infusion (National Institute for Health and Care Excellence 2017).

Starting patients on APO infusion require input from various members of the healthcare team. At our regional specialist movement disorder centre, up until recently, the majority of patients were initiated on a day-case basis according to an established protocol which was implemented by an Advanced Therapy Specialist Nurse within the hospital team under the guidance of a neurologist with additional support, when available, from APO nurses, a service provided by the manufacturer of one of the commercially available apomorphine therapies, Britannia Pharmaceuticals Ltd.

However, as a result of the restrictions in place due to the COVID-19 pandemic, access to hospital day-case facilities was not available, and face-to-face appointments were restricted. There were long waiting lists to start APO infusion treatment and other device-aided therapies were unavailable due to the need for surgery or other procedures. These challenges required a rethink and change of process at Salford to ensure advanced PD patients could receive the treatment they urgently needed to provide them with an acceptable quality of life.

To avoid significant delays in patients receiving treatment and the consequent exacerbation of debilitating PD symptoms, it was decided to undertake initiation of APO infusion therapy in the patient’s own home with assistance from APO nurses. This article reports our experience with APO infusion home initiation, and shares best practice with this new protocol and innovative way of working.

## Materials and methods

We retrospectively reviewed data on our cohort of patients who underwent home APO infusion therapy (APO-go®, Britannia Pharmaceuticals Limited) initiation between June 2020 and June 2022. Duration of treatment and outcomes were assessed up to December 2022. No institutional review board or ethics committee approval was necessary for this work as this was a clinical service evaluation. Informed patient consent was not required as the data were analyzed by members of the treating clinical team (CK, LPS).

Patients were selected for APO infusion therapy based on the consensus suitability criteria published for pump therapy (Trenkwalder et al. 2015). Core criteria for pump therapy included significant ‘off’ periods or dyskinesia limiting further increases in oral treatments, including those deemed suitable for other device-aided therapies not available due to the pandemic restrictions. Suitability for home initiation was determined in each case by the neurologist in charge of the patient’s care. Patients with significant hallucinations, orthostatic symptoms, or other comorbidities felt to be unsuitable for home initiation, were excluded.

APO infusion therapy is generally well tolerated but several important precautions are required to ensure it can be initiated safely in the patient’s home in a one-day procedure, so a specific home initiation protocol was developed and followed for each patient in this analysis (see Table 1). Nurses entering patients’ homes also observed strict COVID-19 safety precautions. Pre-treatment with domperidone is recommended to avoid nausea and in accordance with MHRA guidance (Medicines and Healthcare Products Regulatory Agency 2014), an ECG was performed at baseline to exclude prolonged QTc interval (> 450 ms in men and > 470 ms in women). A baseline full blood count, reticulocyte count, and a Coombs test to assess the possibility of treatment-related haemolytic anaemia were also undertaken, in addition to monitoring for postural hypotension at regular intervals throughout the initiation procedure. ECG and blood tests were performed either in the outpatient clinic or in the general practitioner’s office, with the results reviewed by the treating consultant (CK) prior to treatment initiation.

**Table 1:** Salford protocol: apomorphine (APO) subcutaneous infusion for Parkinson’s disease—guidance for initiating therapy in the patient’s home

Suitability for home initiation	Suitability for home initiation of APO infusion should be determined by the consultant in charge of the patient’s care
APO nurses—actions prior to initiation of APO infusion	Referral is made to APO nurses in writing requesting support for home initiation of APO infusion. An APO nurse will then arrange the following: Order the APO infusion pump; request prescription for infusion lines; District Nurse referral and training on infusion pump, if required; initiation of therapy and titration of APO dose under the guidance and instruction of consultant or hospital’s Advanced Therapy Specialist Nurse Demonstration of the pump and infusion setup procedure for patient/carer; provide support and advice for patient/carer in relation to the infusion pump, skin management and prescriptions; assessment of therapy outcomes and regular communication with the Parkinson’s team
Domperidone pre-treatment*	Pre-treatment with domperidone (10 mg TDS for at least 48 h prior to APO initiation) is recommended to avoid nausea MHRA guidance requires ECG at baseline to exclude prolonged QTc interval (> 450 ms in men and > 470 ms in women) The GP or referring centre should arrange for the patient to have an ECG and if there is no prolonged QTc interval, domperidone should be prescribed
Baseline blood tests	Prior to starting APO infusion treatment, the GP or referring centre should arrange baseline blood tests: full blood count, reticulocytes and Coombs’ test (due to the possibility of treatment-related haemolytic anaemia)
Blood pressure monitoring	Monitoring of postural hypotension should be undertaken regularly before and during the initiation procedure. Baseline lying (or sitting, if lying not possible) and standing blood pressure measurements should be recorded
Pump programming	Any programming of the pump and adjustments to dose should be performed by an APO nurse who is experienced with the pump and dosing of the drug. Both the mg/h dose and the flow rate in ml/h should be recorded
Therapy initiation	At this stage the patient should have a sufficient quantity of APO, infusion lines, and domperidone to last at least 2 weeks. They will also have received a preparation tray along with their pump to aid the infusion setup procedure Providing blood pressure measurements are satisfactory, the APO initiation (using pre-filled syringes of a concentration of 50 mg/10 ml) should be commenced at a rate of 2 mg/h (flow rate 0.4 ml/h) After commencing the infusion, lying (or sitting) and standing blood pressure measurements should be obtained every 15 min for the first hour of infusion and subsequently every half hour for the second hour. Any abnormalities or concerns regarding blood pressure should be reported to the Advanced Therapy Specialist Nurse or consultant in charge of the patient’s care Any hallucinations or adverse effects occurring during the initiation procedure should be reported to the Advanced Therapy Specialist Nurse or consultant in charge of the patient’s care The consultant in charge will provide parameters for dose escalation and reduction of other dopaminergic medications, allowing changes to be made in the community. Changes out with these limits will be discussed by communication between the Advanced Therapy Specialist Nurse and treating consultant
Post-initiation	A letter should be sent to the GP confirming that APO infusion therapy has commenced. The GP will be asked to add APO and related items to the monthly repeat prescription
Follow-up, monitoring, and dose adjustment	Regular home visits will be arranged by the APO nurse to support the patient and to assess therapy outcomes Reports will be sent on a regular basis to the consultant and Advanced Therapy Specialist Nurse in charge of the patient’s care to keep them informed of the patient’s progress All patients are given contact details for the APO nurse, their Hospital Parkinson’s team, and a contact number for the manufacturer’s 24-h helpline for any pump technical problems Regular follow-up appointments (telephone or face-to-face) by the specialist Parkinson’s team should be made to assess the patient’s symptom control The patient can be referred back to their local hospital’s Advanced Therapy Specialist Nurse for follow-up care and medication management if the Nurse and patient are in agreement with this Any adjustments to existing medication or the APO dose will be made depending on the needs of the patient. These changes can be made by the APO nurse under the guidance and instruction of the consultant or Advanced Therapy Specialist Nurse Most Parkinson’s patients commencing APO will need some adjustment of existing medication to achieve optimal symptom management. Requested changes will be carried out by the APO nurse and confirmed in writing to the Parkinson’s team and the GP APO nurses have Honorary Contracts with the Trust. They are not prescribers but act on written instruction of the prescribing consultant or hospital’s Advanced Therapy Specialist Nurse

*APO* apomorphine; *ECG* electrocardiogram; *GP* general practitioner; *MHRA* medicines and healthcare products regulatory agency; *PD* Parkinson’s disease; *TDS* three times daily

*Domperidone has been associated with a small risk of QTc interval prolongation (European Medicines Agency [EMA] 2014). As a precaution, ECGs should be carried out prior to and during APO initiation, and domperidone used at the lowest effective dose for the shortest possible duration (usually < 1 week) according to EMA recommendations

The efficacy of APO infusion therapy during the titration process was assessed by patient self-report and any adverse events were reported to the Advanced Therapy Specialist Nurse or consultant. In some cases, a wearable Parkinson’s KinetiGraph (PKG; Global Kinetics Corporation, Melbourne, Australia) was used by patients to support dose changes and to assess clinical benefits and any adverse effects. The PKG measures movement accelerations of the wrist, providing objective information on bradykinesia and dyskinesia which has been shown to closely correlate with Unified Parkinson's Disease Rating Scale (UPDRS) motor scores (Griffiths et al. 2012).

Post-APO initiation, regular home visits by the APO nurse were arranged to support the patient and also assess outcomes of APO infusion treatment. They subsequently submitted updated reports to the neurologist and Advanced Therapy Specialist Nurse at our centre in charge of the patient’s care who assessed whether any further action was needed and guided further titration of APO dose and modification of other dopaminergic therapies.

## Results

During the specified time period, 27 PD patients were selected as the suitable candidates for home initiation with APO infusion. Demographic characteristics of the patient cohort are shown in Table 2. APO infusion was initiated according to the Salford home initiation protocol described earlier. All patients had caregivers or partners. Each patient was reviewed independently, and medication dosing titrated up/down dependent upon patient and clinician feedback regarding efficacy and tolerability. Changes in flow rate were typically made on a weekly basis, with concomitant reductions in other dopaminergic medications, although this varied according to other factors.

**Table 2:** Demographics and treatment outcomes of the APO infusion home initiation cohort

Variable	APO infusion (n = 27)
Gender (*n*, %)	
Male	13 (43.3%)
Female	14 (56.7%)
Age (years)	63.9 ± 9.1
	(range 39–77)
Treatment duration (days)	365 ± 264
Outcome (*n*)	
Therapeutic response	21
Withdrew from treatment	5
Failed titration	1
Infusion start rate (median, mg/h)	1.0 (range 0.75–2.0)
Infusion maximum rate (median, mg/h)	4.0 (range 1.0–6.0)
24-h infusion used	7/27
Maximum night-time infusion rate (median, mg/h)	2.5 (range 1.0–4.0)
PKG used (n, %)	10/27 (37%)
Oral/transdermal medication reduced (*n*, %)	22/27 (81.4%)

Data are mean ± SD unless otherwise stated

Of the 27 patients initiated onto APO infusion, 21 (77.8%) achieved a therapeutic response. Mean (± SD) duration of treatment was 365 ± 264 days. PKG was used for motor response assessment in 10/27 patients (37%). There was one failed titration, and four patients withdrew from treatment (drowsiness 2, poor response 1, skin nodules 1), whilst one died of pneumonia shortly after starting treatment; this was not deemed related to the treatment. The median initial infusion rate was 1.0 mg/h (range 0.75–2.0) and the median maximum infusion rate was 4.0 mg/h (range 1.0–6.0). Seven of the 27 patients used APO infusion at night as well as during the waking day. A total of 22/27 (81.4%) of patients were able to reduce their oral/transdermal medication use once established on APO infusion therapy.

## Discussion

The change in the APO initiation procedure used at Salford from a hospital-based to home-based setting arose from the need to radically change clinical practice to meet patients’ needs during the COVID-19 pandemic. The significant impact that the pandemic has had on patients with PD treated with device-aided therapies has been recognized around the world (Fasano et al. 2020). The relative complexity of these treatments compared with oral therapies presented a substantial management challenge for movement disorder centres working within the COVID-19 restrictions in place at that time. However, the urgent need to treat and maintain the welfare of PD patients offered an opportunity to rethink the approach to care delivery and implement telemedicine and other initiatives for remote management and monitoring (Fasano et al. 2020).

The success of the initiative in Salford was due to effective collaborative practice, good teamwork, and resourcefulness in the face of the National Health Service (NHS) financial and capacity pressures that were in place during the pandemic. Our results show that home initiation of APO infusion can be undertaken successfully and has benefits for both patients and healthcare teams, in accordance with the license for this product. Home initiation resulted in a significant reduction in the waiting list for APO infusion and waiting time for initiation, as well as allowing day-case beds to be prioritized for admissions for other device-aided therapies. A focus on initiation in the home rather than the hospital setting, and the collaborative working partnership with the APO nurses, also released the hospital’s Advanced Therapy Specialist Nurse capacity to undertake more movement disorder clinics and thereby reduced the clinic waiting list. Patient expectations of treatment and follow-up appointments can be managed and supported by a collaborative and shared team effort comprising APO nurses, NHS specialist nurses, and clinicians.

Our findings are in line with a recent large, prospective, longitudinal study of home APO infusion initiation in 106 PD patients, in which a more rapid improvement in quality of life and autonomy was seen compared to 38 in-hospital admissions (Zagnoli et al. 2023). The costs in this study were reduced in the home initiation group with safety outcomes the same between groups, again supporting the safety of home-based initiation in selected patients. The safety profile of APO in our cohort was similar to previous reports, in which infusion site reactions and drowsiness were the most common adverse effects prompting discontinuation (Katzenschlager et al. 2021).

We acknowledge limitations to this work. First, this was a retrospective analysis and therefore no formal outcome measures were recorded. Second, limitations on nurse autonomy to make dose changes may limit the introduction of such protocols in some healthcare settings. However, as described in Table 1, the treating team provided clear parameters for dose escalation and other changes at the time of treatment initiation.

Due to the success of the home initiation process in place during the pandemic, this will now continue as a standard of care, with day-case admission being reserved for cases not felt suitable for home initiation. It is hoped that our experience and practices with home initiation of APO infusion will encourage other centres to implement similar procedures.

## Data Availability

The datasets generated during and/or analyzed during the current study are available from the corresponding author on reasonable request.
